# Proteomic Approach to Study the Effect of *Pneumocystis jirovecii* Colonization in Idiopathic Pulmonary Fibrosis

**DOI:** 10.3390/jof11020102

**Published:** 2025-01-29

**Authors:** Jonás Carmona-Pírez, Rocío Salsoso, Eléna Charpentier, Cinta Olmedo, Francisco J. Medrano, Lucas Román, Carmen de la Horra, Yaxsier de Armas, Enrique J. Calderón, Vicente Friaza

**Affiliations:** 1 Instituto de Biomedicina de Sevilla, Hospital Universitario Virgen del Rocío/Consejo Superior de Investigaciones Científicas/Universidad de Sevilla, 41013 Seville, Spain; jonas.carmona.sspa@juntadeandalucia.es (J.C.-P.); msalsoso@us.es (R.S.); fmedrano@us.es (F.J.M.); cdlhorra-ibis@us.es (C.d.l.H.); vfriaza-ibis@us.es (V.F.); 2 Research Network on Chronicity, Primary Care, and Health Promotion (RICAPPS), Institute of Health Carlos III (ISCIII), 28029 Madrid, Spain; 3 Subdirección Técnica Asesora de Gestión de la Información, Servicio Andaluz de Salud (SAS), 41071 Seville, Spain; 4 Departamento de Medicina, Hospital Universitario Virgen del Rocío, Facultad de Medicina, Universidad de Sevilla, 41009 Seville, Spain; 5 Unidad Médico-Quirúrgica de Enfermedades Respiratorias, Hospital Universitario Virgen del Rocío, 41013 Seville, Spain; mcinta.olmedo.sspa@juntadeandalucia.es (C.O.); lucroman91@gmail.com (L.R.); 6 Centro de Investigación Biomédica en Red de Epidemiología y Salud Pública, 28029 Madrid, Spain; 7 Departments of Clinical Microbiology Diagnostic and Pathology, Hospital Center of Institute of Tropical Medicine “Pedro Kourí”, Havana 11400, Cuba; yaxsier2017@gmail.com; 8 Departamento de Microbiología y Patología, Instituto de Patología Infecciosa y Experimental “Francisco Ruiz Sánchez”, Guadalajara 44100, Mexico

**Keywords:** idiopathic pulmonary fibrosis, proteomics, *Pneumocystis jirovecii*, *Pneumocystis* colonization, iTRAQ quantification, protein–protein interaction networks

## Abstract

Idiopathic pulmonary fibrosis (IPF) is a chronic, progressive, and interstitial disease with an unclear cause, believed to involve genetic, environmental, and molecular factors. Recent research suggested that *Pneumocystis jirovecii* (PJ) could contribute to disease exacerbations and severity. This article explores how PJ colonization might influence the pathogenesis of IPF. We performed a proteomic analysis to study the profile of control and IPF patients, with/without PJ. We recruited nine participants from the Virgen del Rocio University Hospital (Seville, Spain). iTRAQ and bioinformatics analyses were performed to identify differentially expressed proteins (DEPs), including a functional analysis of DEPs and of the protein–protein interaction networks built using the STRING database. We identified a total of 92 DEPs highlighting the protein vimentin when comparing groups. Functional differences were observed, with the glycolysis pathway highlighted in PJ-colonized IPF patients; as well as the pentose phosphate pathway and miR-133A in non-colonized IPF patients. We found 11 protein complexes, notably the JAK-STAT signaling complex in non-colonized IPF patients. To our knowledge, this is the first study that analyzed PJ colonization’s effect on IPF patients. However, further research is needed, especially on the complex interactions with the AKT/GSK-3β/snail pathway that could explain some of our results.

## 1. Introduction

Idiopathic pulmonary fibrosis (IPF) is a chronic, progressive, and eventually fatal interstitial lung disease (ILD) characterized by the formation of excessive fibrous tissue in the lungs, that occurs principally in older adults. This scarring leads to a progressive decline in pulmonary function, typically presenting with symptoms such as exertional dyspnea and a persistent dry cough [1]. The term “idiopathic” highlights the absence of a known cause for the disease, differentiating it from other forms of pulmonary fibrosis associated with known etiologies such as connective tissue diseases or environmental exposures [2]. Pathologically, IPF is associated with a distinct histological pattern known as usual interstitial pneumonia (UIP), which is distinguished from other ILD patterns by its temporal and spatial heterogeneity. While IPF is often discussed as a singular condition, it is important to acknowledge variations within its clinical spectrum, potentially related to differences in genetic predispositions or environmental factors [3].

The precise cause of IPF remains elusive, but its pathogenesis is thought to involve a complex interplay of genetic, environmental, and molecular factors. It is widely accepted that aberrant wound healing and persistent alveolar epithelial injury play a central role. Repeated micro-injuries to the alveolar epithelium lead to an abnormal repair process, characterized by the excessive activation of fibroblasts and myofibroblasts, ultimately resulting in the deposition of extracellular matrix (ECM) components, particularly collagen. Over time, this excessive ECM deposition compromises lung architecture and function. Genetic mutations, such as those in genes involved in telomere maintenance, have been implicated in familial cases of IPF, while environmental exposures, such as cigarette smoke, air pollution, and certain occupational hazards, may contribute to disease onset. Epigenetic modifications, oxidative stress, and dysregulated immune responses are also thought to play roles in the fibrotic process [2,4].

The potential involvement of infectious agents in the pathogenesis of IPF has been the subject of increasing interest. Several viral infections, particularly those caused by herpesviruses, including Epstein–Barr virus (EBV) and cytomegalovirus (CMV), have been investigated as potential triggers for the initiation or exacerbation of pulmonary fibrosis. These viruses can cause chronic inflammation and alveolar epithelial cell damage, which may contribute to the repetitive injury that is characteristic of IPF [5]. In addition, bacterial and fungal pathogens may exacerbate lung injury in susceptible individuals by inducing immune dysregulation [6]. However, whether infectious agents serve as primary causative factors or merely act as cofactors in disease progression remains an open question. Recent studies suggest that, while infectious agents may not directly cause IPF, they could play a significant role in disease exacerbations and influence disease severity through modulation of the host immune response. The presence of *Pneumocystis jirovecii* (PJ) colonization has been described in chronic lung diseases, including idiopathic pulmonary fibrosis [7,8]. Several studies have started to explore the molecular and immune responses triggered by PJ colonization in the context of chronic pulmonary diseases. These investigations indicate a complex interplay between the fungus and the host’s immune system, suggesting that various immune dysregulations might exacerbate lung injury and promote fibrosis [9,10,11].

This article explores the potential influence of PJ colonization on the pathogenesis of IPF, highlighting both their direct and indirect effects on lung health. We performed a proteomic analysis to identify differences in the protein profile of IPF and control patients, with and without PJ colonization.

## 2. Materials and Methods

### 2.1. Participants

Participants were recruited from the Respiratory Endoscopy Unit at the Virgen del Rocio University Hospital (Seville, Spain). All participants completed written informed consent and, according to our hospital’s regulations, the procedure for requesting and authorizing research studies was completed. The diagnosis of IPF was made by lung biopsy in all cases. The control group was patients with mild hemoptysis without malignancy. Participants were also divided into colonized or non-colonized PJ groups based on the results of PCR analysis. The exclusion criteria were underlying autoimmune disorders, malignant disease, immunosuppressive therapy (cytotoxic and steroids treatment), positive status for human immunodeficiency virus, and any clinical symptom and/or radiological evidence that could correspond to pneumonia. Bronchoalveolar lavage (BAL) samples were collected from selected patients for further analysis. The characteristics of the study population are described in Table 1.

### 2.2. Sample Collection

Bronchoalveolar lavages were conducted following standard procedures. Briefly, sterile saline solution (0.9% *w*/*v*) was instilled under local anesthesia, with a fiberoptic bronchoscope (Type 40; Olympus, Tokyo, Japan) and recovered immediately by suction. About 4 mL of each BAL sample were transported immediately to the laboratory in a sterile refrigerated container where they were aliquoted in two aliquots of 250 μL for *Pneumocystis* detection and the rest of the sample was used for the proteomic study.

### 2.3. Detection of Pneumocystis jirovecii

Whole BAL aliquots were digested with proteinase K at 56 °C with 10 mM of EDTA and 0.5% of SDS over 4 h and total DNA was extracted and purified using Nucleospin Tissue Kit (Macherey-Nagel, Duren, Germany). The presence of *Pneumocystis* was analyzed by nested PCR amplification of the *Pneumocystis* mtLSU rRNA gene as described elsewhere [12]. Patients whose PJ DNA was detected by nested PCR in both BAL aliquots and in two independent analyses were considered positive by PJ. While patients whose samples, following the same criteria, were always negative were considered non-colonized patients. To prevent false positives from contamination, pipettes with filters were used in all stages. DNA extraction, preparation of the reaction mixture, PCR amplification, and detection were performed in different areas. To detect any cross-contamination, all PCR was performed with a negative control of sterile water. Each experiment was performed at least twice.

### 2.4. iTRAQ Analysis

An untargeted proteomic analysis with 4-plex isobaric tags for relative and absolute quantification (iTRAQ) was performed to identify differences in the protein profile of IPF and control patients with and without PJ.

Analysis was performed in five phases: extraction and protein depletion, trypsin digestion and iTRAQ labeling, high pH reversed-phase fractionation, liquid chromatography–mass spectrometry nanoLC nanoESI-QTOF (QSTAR^®^ XL Hybrid LC/MS/MS System, Applied Biosystems) and MS data analysis using Protein Pilot software 5.0.2 (AB Sciex).

Briefly, BAL fluid samples were centrifuged at 800× *g* for 10 min, and the supernatants were collected. Sample protein concentration was measured by Braford assay and a similar quantity of proteins was added from each sample to produce the different pools. Samples from patients with IPF, with and without PJ, were pooled separately. The supernatants were concentrated with Amicon^®^ Ultra 0.5 mL 3 k Ultracell (Merk Millipore, Milan, Italy). Subsequently, six volumes of cold acetone were added to the concentrated supernatants and incubated for 4 h at −20 °C. The mixture was centrifuged at 14,000× *g* for 10 min, and the resulting pellets were resuspended in a protein solubilization buffer.

For further sample preparation, albumin and IgG were depleted using the ProteoPrep^®^ Immunoaffinity Albumin and IgG Depletion Kit (Sigma-Aldrich). The resulting proteins were precipitated using a methanol–chloroform method and resuspended in 1% SDS at a final concentration of 5 mg/mL. Subsequently, 100 µg of peptides from each group were reduced, cysteine-blocked, and digested with trypsin. The digested peptides were labeled with iTRAQ reporters 114, 115, 116, and 117, according to the manufacturer’s instructions (Applied Biosystems). The reference was assigned to a patient with mild hemoptysis without *Pneumocystis* labeled as 114-iTRAQ.

The contents of all iTRAQ reagent-labeled sample tubes were combined into one tube to perform the LC-MS/MS analysis. We performed a comparative analysis to identify differentially expressed proteins (DEPs) by applying a Student’s *t* test to detect expression changes, considering downregulation and upregulation a fold change of 0.8 and 1.2, respectively, and *p*-value ≤ 0.05. All data were analyzed using Protein Pilot software (AB Sciex) and MASCOT v2.1 software (Matrix Science) and were searched against the NCBInr database.

### 2.5. Pathway and Process Enrichment Analysis

We performed a pathway and process enrichment analysis for each given gene list, in the following ontology sources: GO Biological Processes, Reactome Gene Sets, KEGG pathways, WikiPathways, and MIRNA. We used the R package gProfiler2 [13] and the g:Profiler—interoperable web service [14] to perform this analysis. Only genes with at least one annotation in the genome were used as the enrichment background. We applied the g:SCS method [14] for computing multiple testing corrections for *p*-values gained from GO and pathway enrichment analysis. The experiment-wide threshold was a = 0.05 and the user threshold *p*-value = 0.05. The g:SCS method also pre-calculates a threshold t for list sizes up to 1000 genes. It corresponds to the 5% upper quantile of randomly generated queries of that fixed input size. The resulting *p*-values are corrected by multiplying them by the initial experiment-wide threshold a = 0.05 and the ratio of the threshold t.

### 2.6. Protein–Protein Interaction Networks

We built a protein–protein interaction network for each gene list using the STRING database [15]. Only physical interactions (physical score > 0.150) were included. Genetic interactions were not included because they capture functional relationships that could be like those in Gene Ontology and we aim to detect protein complexes. The resultant three networks were analyzed with the Cytoscape StringApp v3.7.2 [16] and the clusterMaker Cytoscape plugin [17]. The Molecular Complex Detection (MCODE) algorithm [18] was applied to identify densely connected network clusters. The algorithm has three steps:

(1) Weighting: It is proportional to the interconnectivity between nodes; those more interconnected nodes will have a higher score;
(2) Complex prediction: It orders the nodes based on the weighting, starting with the node with the highest weight and iteratively moving out. Then, it adds nodes to the complexes considering a given threshold;
(3) Filtering: Hair and fluff filters are applied to improve the quality of the clusters.

The MCODE method was specifically designed to find protein complexes in PPI networks [18], and we set its parameters to optimize results accuracy (depth = 100; node score% = 0; haircut = true; fluff = false; percentage for complex fluffing = 0.2) [19]. Once we obtained the protein complexes, we performed a pathway and process enrichment analysis as described previously. The complexes obtained were then named taking into consideration the best-scoring terms and the consensus of all researchers.

All the bioinformatics analyses were conducted using RStudio software (R version 4.3.3, RStudio, Boston, MA, USA), Cytoscape software (version 3.10.2, Institute for Systems Biology, Seattle, WA, USA), and interoperable web resources g:Profiler [14] and STRING [15].

## 3. Results

### 3.1. iTRAQ-Based Proteomics Analysis to Identify DEPs

**Figure 1:**
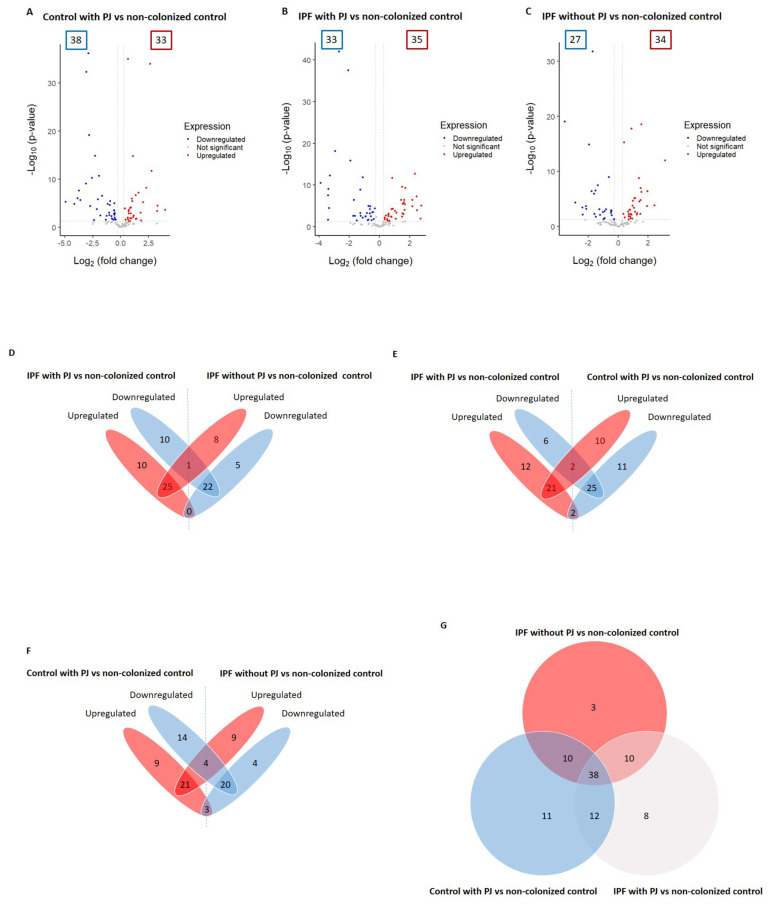
Differentially expressed proteins (DEPs) associated with the groups: control with *Pneumocys jirovecii* (PJ), idiopathic pulmonary fibrosis (IPF) colonized by PJ, and IPF non-colonized by PJ (**A**–**C**). The DEPs were identified by comparing to the control group non-colonized by PJ; the comparison results are presented as volcano plots. The protein expression differences with a fold change of 0.8 or 1.2 (cutoff line, x = 0.260344) were considered significant at a *p* value of 0.05 (cutoff line, y = 1.30). The numbers in the colored frames indicate the identified DEP numbers. The dots that are labeled with protein designations represent some DEPs in the study. (**D**–**F**) Overlapping DEPs were analyzed, and the shared protein numbers between the groups are shown as a Venn diagram. (**G**) Numbers of shared proteins among the groups are shown as a Venn diagram.

We identified a total of 92 proteins differentially expressed among control with PJ, IPF with PJ, and IPF without PJ groups (please see Table 2 and Table S1 in the Supplementary Materials file). Among them, 71 DEPs were associated with the control group with PJ, including 38 downregulated and 33 upregulated proteins (Figure 1A and Table 2). We identified 68 DEPs in the IPF with PJ group of which 33 were downregulated and 35 upregulated (Figure 1B and Table 2). Regarding the IPF without PJ group, we identified 61 DEPs, 27 downregulated, and 34 upregulated (Figure 1C and Table 2).

We detected that only one protein (immunoglobulin J chain) was downregulated in the IPF patients with PJ and upregulated in IPF patients without PJ. With no proteins regulated in the opposite direction between these two groups (Figure 1D and Table S1). Regarding the comparison between the IPF group with PJ and the control group with PJ, we found that two proteins (hemoglobin subunits alpha and beta) were downregulated in the first group and upregulated in the second one, while tyrosine 3-monooxygenase and vimentin were regulated in opposite directions (Figure 1E and Table S1). Similarly, this occurred in the comparison between IPF patients without PJ and controls with PJ, in which we identified hemoglobin subunits alpha and beta, and keratin 1 as downregulated in the IPF group and upregulated in the control group with PJ, while the proteins phosphoglycerate kinase 1, alpha-1-antitrypsin, pulmonary surfactant-associated protein B and vimentin were regulated in antagonistic directions (Figure 1F and Table S1).

To summarize these results, Table 3 shows DEPs with opposite changes with significant *p*-values in the three groups, showing that only three proteins fulfilled these requirements: vimentin and hemoglobin subunits alpha and beta.

As our objective was to explore the mechanisms of PJ in patients with and without IPF, our next step was the functional analysis of the three groups under study.

### 3.2. Pathway and Process Enrichment Analysis

**Figure 2:**
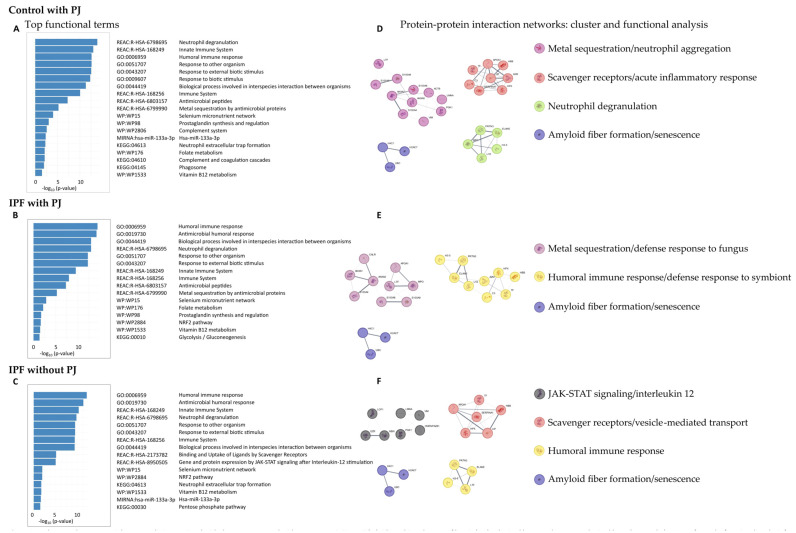
Pathway and process enrichment analysis associated with the groups: control with *Pneumocystis jirovecii* (PJ), idiopathic pulmonary fibrosis (IPF) colonized by PJ, and IPF non-colonized by PJ (**A**–**C**). We performed a functional analysis for each given gene list, in the following ontology sources: GO Biological Processes, Reactome Gene Sets (REAC), KEGG pathways, WikiPathways (WP) and MIRNA. It includes up to 5 terms in each category and ordered by −log10 *p*-value. We built a protein-protein interaction network for each gene list (respectively, (**D**,**E**,**F**)) using the STRING database. We applied the Molecular Complex Detection (MCODE) algorithm to identify densely connected protein complexes. Then, we performed a functional analysis as described previously. The complexes found were named considering the best-scoring terms and the consensus of all researchers.

The functions of DEPs in the control group with PJ, the IPF group with PJ, and the IPF group without PJ were explored using GO Biological Processes, Reactome Gene Sets, KEGG pathways, WikiPathways, and microRNA terms (Figure 2).

In the control group with PJ, we obtained 108 enriched GO terms for Biological Processes, 22 Reactome terms, 3 KEGG pathways, 6 WikiPathways, and 1 microRNA term (Table S2). The top terms for each category are shown in Figure 2A, highlighting the GO Biological Processes terms humoral immune response (GO:0006959), response to other organism (GO:0051707), and hydrogen peroxide catabolic process (GO:0042744); the Reactome Gene Set terms defined as neutrophil degranulation (R-HSA-6798695), innate immune system (R-HAS-168249), metal sequestration by antimicrobial proteins (R-HSA-6799990), binding and uptake of ligands by scavenger receptors (R-HSA-2173782), and surfactant metabolism (R-HSA-5683826); the KEGG pathway terms neutrophil extracellular trap formation (KEGG:04613) and complement and coagulation cascades (KEGG:04610); the WikiPathways selenium micronutrient network (WP:WP15), prostaglandin synthesis and regulation (WP:WP98), folate metabolism (WP176), and vitamin B12 metabolism (WP1533); and the microRNA term hsa-miR-133a-3p

Regarding the IPF group with PJ, we detected 82 enriched GO terms for Biological Processes, 17 Reactome terms, 1 KEGG pathway, and 5 WikiPathways terms (Table S3). No microRNA term was identified. The top terms are shown in Figure 2B. GO Biological Processes terms obtained were very similar to those identified in the other groups, highlighting humoral immune response (GO:0006959), and biological process involved in interspecies interaction between organisms (GO:0044419). Regarding the Reactome Gene Set terms, we detected some differences compared to the control group with PJ and similarities to the IPF group without, highlighting the terms gene and protein expression by JAK-STAT signaling after interleukin-12 stimulation (R-HSA-8950505) and interleukin-12 signaling (R-HSA-9020591). We identified only one KEGG term, glycolysis/gluconeogenesis (KEGG:00010). Finally, the WikiPathways found were selenium micronutrient network (WP15), prostaglandin synthesis and regulation (WP98), folate metabolism (WP176), vitamin B12 metabolism (WP1533), highlighting the term NRF2 pathway (WP2884).

In the IPF group without PJ, we identified 55 enriched GO terms for Biological Processes, 16 for Reactome Gene Sets, 3 for KEGG pathways, 3 for WikiPathways terms, and 1 for microRNA term (Table S4). The top terms for each category are shown in Figure 2C; the results for GO Biological Processes were like those found in the other groups, standing out the terms humoral immune response (GO:0006959) and response to other organisms (GO:0051707). Regarding the Reactome Gene Set terms, the results were like those obtained in the IPF group with PJ, highlighting the terms gene and protein expression by JAK-STAT signaling after interleu-kin-12 stimulation (R-HSA-8950505) and interleukin-12 signaling (R-HSA-9020591). Among the KEGG pathways, the terms neutrophil extracellular trap formation (KEGG:04613) and pentose phosphate pathway (KEGG:00030) were highlighted. The significant WikiPathway terms were selenium micronutrient network (WP15), NRF2 pathway (WP2884), and vitamin B12 metabolism (WP1533), like those obtained in the IPF group. Finally, we also identified the microRNA term hsa-miR-133a-3p.

### 3.3. Protein–Protein Interaction (PPI) Networks: Cluster and Enrichment Analysis

The PPI network from the control group with PJ consisted of 62 nodes and 185 edges (Figure S1), and we identified four protein communities (Figure 2D), which we referred to as metal sequestration/neutrophil aggregation, scavenger receptors/acute inflammatory response, neutrophil degranulation, and amyloid fiber formation/senescence complexes.

The metal sequestration/neutrophil aggregation complex was characterized by metal sequestration by antimicrobial proteins (R-HSA-6799990), neutrophil aggregation (GO:0070488), sequestering of zinc ion (GO:0032119), prostaglandin synthesis and regulation (WP98) and vitamin D receptor pathway (WP2877). It included the proteins ACTB, ANXA2, ANXA5, LMNA, LTF, PGK1, S100A4, S100A6, S100A8, S100A9 and VIM. The scavenger receptors/vesicle-mediated transport complex was characterized by the terms scavenging of heme from plasma (R-HSA-2168880), acute inflammatory response (GO:0002526), binding and uptake of ligands by scavenger receptors (R-HSA-2173782) and post-translational protein phosphorylation (R-HSA-8957275); it included the proteins A2M, APOA1, C3, HBB, HP, HPX, SERPINA1 and TF. The neutrophil degranulation complex was defined by the terms antimicrobial peptides (R-HAS-6803157), neutrophil degranulation (R-HSA-6798695), response to yeast (GO:0001878), and transcriptional misregulation in cancer (KEGG:05202); it included the proteins ELANE, H3-5, MPO, LYZ, and PRTN3. Finally, the amyloid fiber formation/senescence complex was defined by the terms amyloid fiber formation (R_HSA-977225), senescence-associated secretory phenotype (R_HSA_2559582), and oxidative stress-induced senescence (R-HAS-2559582), and it was formed by the proteins H2AC7, H4C1, and UBC.

The PPI network from the IPF group with PJ consisted of 53 nodes and 133 edges (Figure S2), and we identified 3 protein communities (Figure 2E) which we referred to as metal sequestration/defense response to fungus, humoral immune response/defense response to symbiont, and amyloid fiber formation/senescence complexes.

The metal sequestration/defense response to fungus complex was characterized by the terms metal sequestration by antimicrobial proteins (R-HSA-6799990), defense response to fungus (GO:0050832), chemotaxis (GO:0006935), and taxis (GO:0042330); it was formed by the proteins ANXA1, ANXA2, APOA1, CALR, LTF, MPO, S100A4, S100A8, and S100A9. The humoral immune response/defense response to symbiont complex was defined by the terms humoral immune response (GO:0006959), defense response to symbiont (GO:0140546), and neutrophil degranulation (R-HAS-6798695); it was composed by the proteins A2M, C3, ELANE, HBB, HPX, H3-5, LYZ, PRTN3, and TF. Finally, the amyloid fiber formation/senescence complex was the same as previously described in the PPI network from the control group with PJ.

Finally, the PPI network from the IPF group without PJ was integrated by 50 nodes and 117 edges (Figure S3), and we detected 4 protein communities (Figure 2F), which we named JAK-STAT signaling/interleukin 12, scavenger receptors/vesicle-mediated transport, humoral immune response, and amyloid fiber formation/senescence complexes.

The JAK-STAT signaling/interleukin 12 complex was characterized by the terms gene and protein expression by JAK-STAT signaling after IL 12 stimulation (R-HSA-8950505), IL12 signaling (R-HAS-9020591), and membrane-to-membrane docking (GO:0022614), among others; being formed by the proteins EZR, HNRNPA2B1, LCP1, LMNA, MSN, PGK1, and VIM. The scavenger receptors/vesicle-mediated transport protein complex was like that identified in the control group with PJ and defined by the terms scavenging of heme from plasma (R-HSA-114608), vesicle-mediated transport (R-HSA-5653656) and binding and uptake of ligands by scavenger receptors (R-HSA-2173782); it included the proteins APOA1, HBB, HP, HPX, SERPINA1 and TF. The humoral immune response complex was characterized by the terms antimicrobial humoral response (GO:0019730), antimicrobial peptides (R-HSA-6803157), humoral immune response (GO:0006959) and systemic lupus erythematosus (KEGG:05322), being similar to that obtained in the IPF group with PJ; it was formed by the proteins ELANE, H3-5, LTF and PRTN3. Finally, the amyloid fiber formation/senescence complex was the same as previously described.

## 4. Discussion

This study explored the protein expression profiles of IPF and control patients and the effect of PJ colonization on them. We applied an iTRAQ-based method to identify differences in protein profiles and detected relevant DEPs, highlighting the protein vimentin. We performed pathway and process enrichment analyses revealing functional differences between groups, highlighting the glycolysis pathway in patients with IPF colonized by PJ, and the pentose phosphate pathway and microRNA miR-133A in IPF patients non-colonized by PJ. Three protein interaction networks were built, identifying 11 protein complexes in total, highlighting the JAK-STAT signaling complex in IPF patients non-colonized by PJ. To our knowledge, this is the first study to apply a proteomic analysis to study the effect of PJ colonization in IPF patients. Our findings can contribute to improving the knowledge of proteomic biomarkers in IPF patients and the effect of PJ.

The most relevant finding of this study is the identification of vimentin as a DEP when comparing controls colonized by PJ with IPF patients. We also identified the proteins hemoglobin subunits alpha and beta as DEPs in this comparison; however, we must consider that controls were individuals who presented hemoptysis and underwent bronchoscopy without finding lung lesions, and consequently, we consider this result not significant. It is important to note that vimentin presented opposite changes with significant probability values in the three groups; it was downregulated in the control group colonized by PJ and upregulated in IPF patients (colonized and non-colonized). Vimentin is a class-III intermediate filament protein detected predominantly in mesenchymal cells, such as fibroblasts, endothelial cells, macrophages, melanocytes, and lymphocytes. It plays a fundamental role in many cellular processes, including cell adhesion and migration, cell shape and plasticity, organelle anchoring, and signal transduction, with implications for wound healing, embryonic development, immune response, or cancer progression [20]. Circulating anti-vimentin IgG antibodies are considered a potential biomarker in IPF because their levels were found to be much greater in IPF patients and were associated with worse prognosis [21]. Among its functions, vimentin may act as a co-receptor for viral entry, as seen with severe acute respiratory syndrome coronavirus (SARS-CoV-2) [22] and SARS-CoV-2 infections [23,24]. The virus binds to angiotensin-converting enzyme 2 (ACE2), its primary receptor, and the interaction with vimentin facilitates virus internalization [24,25]. In addition, some viruses use vimentin to assemble viral factories and facilitate replication [20]. We know vimentin inhibition reduces SARS-CoV-2 entry and protects cells from virus-induced damage [24]. Infections with Plasmodium, the parasite responsible for malaria, can also affect the expression and function of vimentin [26]. This study in murine models of hepatocellular carcinoma (HCC) has shown that Plasmodium infection suppresses cancer progression and metastasis by inhibiting epithelial–mesenchymal transition (EMT). This inhibition is associated with a decrease in the expression of vimentin and snail, proteins that promote EMT, and an increase in the expression of E-cadherin, a protein that suppresses it. The findings suggested that Plasmodium infection may interact with the protein kinase B (AKT)/glycogen synthase kinase 3 (GSK-3β)/snail signaling pathway, leading to the downregulation of vimentin expression [26]. Vimentin has been proposed as a potential therapeutic target, and drugs that inhibit vimentin, such as withaferin a and simvastatin, have shown promising results in reducing viral entry and tumor progression [20,27]. Our results suggested that vimentin in control patients colonized by PJ had a similar expression pattern to that found in Plasmodium infections; that is, the infection would downregulate vimentin expression. However, this pattern was inconsistent in IPF patients colonized by PJ, where there was a similar upregulated expression of vimentin to those IPF patients non-colonized by PJ. Further analyses of the molecular mechanisms underlying this result are needed to understand it. The complex interactions between PJ and IPF and the AKT/GSK-3β/snail pathway could explain some of these results.

We also found that the glycolysis/gluconeogenesis KEGG term was highlighted in patients with IPF and PJ, a pathway not detected in the functional analysis of IPF patients non-colonized by PJ or in control patients with PJ. The intersection of this pathway in patients with IPF and PJ was formed by the DEPs aldehyde dehydrogenase 2 (ALDH2) (upregulated), aldehyde dehydrogenase 3A1 (ALDH3A1) (downregulated), fructose-bisphosphatase 1 (FBP1) (upregulated) and triosephosphate isomerase 1 (TPI1) (upregulated), and we hypothesize that they could be essential in regulating the interaction with the AKT/GSK-3β/snail pathway and the development of lung fibrosis among these patients. ALDH2 is a mitochondrial enzyme crucial for the detoxification of reactive aldehydes, and plays a cardioprotective role by regulating the AKT/GSK-3b/snail pathway; overexpression of ALDH2 enhances the B-cell lymphoma 2 protein (BCL2), GSK-3β, and AKT levels [28]. ALDH2 interacts with the transforming growth factor beta-1 (TGF-β1), an essential protein in fibrosis, through TGF-β type I receptor/Smad3 signaling. Analysis of lung tissue from IPF patients revealed decreased ALDH2 expression in fibroblasts from IPF lungs compared to control lungs [29]. Our results were not significant for ALDH2 in control patients colonized by PJ and IPF patients non-colonized; however, we hypothesize that ALDH2 could be upregulated in patients colonized by PJ. Regarding the protein ALDH3A1, it was downregulated in all three groups, with similar expression levels in all of them. ALDH3A1 has been described to present high levels in cancer cells and low levels in normal pneumocytes [30]. ALDH3A1 overexpression is associated with the induction of EMT and increased expression of EMT markers such as vimentin, fibronectin, and zinc finger E-box binding homeobox 1 (Zeb1) [31]. The protein FBP1 also had a similar pattern in IPF patients with upregulation independently of PJ and was not significant in controls with PJ. FBP1 is a critical enzyme in gluconeogenesis. In cancer cells, FBP1 is usually inhibited, or its expression is reduced with implications on the EMT [32,33]. This contributes to the well-known “Warburg effect”, where fibroblasts in IPF and cancer cells promote glycolysis, which facilitates their rapid proliferation [34]. It has been studied that loss of FBP1 by snail-mediated repression could provide metabolic advantages in basal-like breast cancers (BLBC); snail, a transcription factor, binds directly to the E-boxes present in the promoter of the FBP1 gene, repressing its transcription by methylation [33]. It has also been shown that methylation of the FBP1 promoter, which can be induced by other pathways such as the nuclear factor-kappa B (NF-κB) pathway, has also been observed in other types of cancer, such as non-small cell lung cancer (NSCLC) [35] and lung adenocarcinoma (LUAD) [32,36,37]. Finally, the TPI1 protein was overexpressed in IPF patients colonized by PJ and was not significant in the other groups. TPI1 is a glycolytic enzyme that is thought to have some relevant roles in cancer. Upregulation of TPI1 has been reported in breast cancer, lung cancer, gastric cancer, and pancreatic cancer [38,39,40,41]. However, the knowledge of TPI1 in cancer is limited and is thought to be related to gene sharing and moonlighting functions [42]. The specific role of TPI1 in lung cancer development is not completely clear but it has been described a significant correlation of TPI1 with poor prognosis, especially in LUAD [39]. It has been studied that TPI promotes breast cancer growth and migration due to the association with the protein cell division cycle associated 5 (CDCA5) which activates the phosphatidylinositol-3-kinase (PI3K) and then the pathways AKT/GSK-3β/snail and mammalian target of rapamycin complex 1 (mTORC1) [38]. We hypothesize that a similar mechanism could be involved in IPF patients and further analyses are needed to study it.

In IPF patients non-colonized by PJ, our analysis highlighted the pentose phosphate pathway (PPP). PPP and glycolysis are closely connected, and both are highly relevant for cancer cells. Among the DEPs in these patients, we found the protein FBP1, previously discussed, which was upregulated in these patients, along with phosphogluconate dehydrogenase (PGD), which was also upregulated, and transaldolase (TALDO1), which was downregulated. FBP1 and TALDO1 are essential proteins controlling the reversible gate between PPP and glycolysis [43]. Regarding TALDO1, previous studies have highlighted its overexpression in head and neck carcinomas, gastric cancer, colorectal cancer, upper tract urothelial carcinoma, ovarian cancer, and hepatocellular carcinoma metastases [44,45,46,47,48,49]. TALDO1 has also been identified as a potential biomarker in NSCLC and was found to be overexpressed in the epithelium of smokers compared to non-smokers [50,51]. TALDO1 is activated by nuclear factor erythroid-2-related factor 2 (NRF2) [52], a promoter of tumor progression, which inhibits EMT by suppressing snail expression during pulmonary fibrosis in mice experiments [53]. The functional analysis also found the NRF2 pathway associated with all IPF patients (PJ-colonized and non-colonized). NRF2 activation seems to inhibit EMT by suppressing snail expression during pulmonary fibrosis, which could reduce oxidative stress and modulate inflammation [53]. There is also evidence that NRF2 overexpression is associated with tumor grade, stage, invasion, and poor overall survival in upper tract urothelial cancer [49,54], which might be related to the catabolic metabolism of cancer cells in the metastatic phase versus an anabolic metabolism in early stages of cancer where nourished microenvironments predominate [43]. Our results were not significant for TALDO1 in PJ-colonized IPF and PJ-colonized control patients. We should be cautious in interpreting TALDO1 results, and further studies are needed to understand the potential role of TALDO1 in IPF patients. Regarding the protein PGD, this protein was upregulated in all IPF patients (colonized and non-colonized by PJ) and was not significant in controls. It has a prominent role in the growth and survival of cancer cells, particularly in LUAD, where it has been studied to inhibit the AMP-Kinase pathway, promoting glycolysis and fatty acid synthesis [55]. The JAK-STAT protein complex detected in IPF patients non-colonized by PJ could also be relevant in the regulation of the AKT/GSK-3β/snail pathway through the phosphoinositide 3-kinases (PI3K) [56]. This protein complex included the protein vimentin and it has been studied that its dysregulation is associated with several cancers and autoimmune diseases [57].

In our study, no patient had cancer, but it is known that persistent IPF increases a patient’s risk of developing lung cancer [34]. Further analysis on ALDH2, ALDH3a1, FBP1, PGD, TALDO1, and TPI1 as biomarkers on the evolution of IPF patients, their association with PJ infection, and their lung cancer risk could be relevant. We also identified microRNA 133A as a functional term that characterized IPF patients non-colonized by PJ. According to our functional analysis, this microRNA is associated with pulmonary surfactant type B, which was upregulated in IPF patients without PJ and downregulated in control patients colonized by PJ, being not significant in IPF patients with PJ. In this sense, the capacity of Pneumocystis to induce selective inhibition of pulmonary surfactant protein B expression and that this down-regulation is mediated at the level of mRNA expression has been shown in animal models [58]. Surfactants and microRNAs are considered potential biomarkers in IPF [21]. Specifically, it has been studied that microRNA 133A seems to inhibit multiple components of TGF-β1 profibrogenic pathways, suggesting a potential therapeutic target [59]; however, its mechanism has not been fully understood. Regarding surfactant B, its serum levels have been considered as a biomarker in IPF; however, only preliminary work in this direction has been published [60]. Our results support these research lines, but further analyses are needed to shed light on them.

Regarding other identified protein complexes, our results showed that protein complexes in control patients colonized by PJ were mainly related to innate immune response. In contrast, IPF patients included a mixture of innate and adaptive immune response protein complexes. We also found that all groups shared the amyloid fiber formation/senescence complex; this result might respond to the aging process in the study population [61]. Control and IPF patients colonized by PJ shared a protein complex related to innate immune response called metal sequestration complex, whereas IPF patients shared a complex related to humoral immune response. IPF is a progressive fibrosing interstitial lung disease in which innate and adaptive inflammatory processes are activated [62]. Fungal pathogens, such as PJ, are thought to cause immune dysregulations and promote pulmonary fibrosis [6], and our results support this hypothesis. However, further analyses are needed to understand the mechanisms fully. It is known that the host immune response is essential in the response to PJ. While immunocompetent hosts are able to eliminate PJ without symptoms, immunodeficient hosts may develop pneumonia with an excessive inflammatory response and lung damage [63]. This is partly reflected in the characteristics of the protein complexes identified in patients colonized by PJ. While control patients (i.e., immunocompetent) showed a milder immune response, IPF patients had a more intense response. However, we have to consider in this interpretation that the network analysis applied was limited to physical interactions and was strict in identifying protein complexes.

In conclusion, this is the first study to apply a proteomic approach to study the effect of PJ colonization in IPF patients. Our findings could be of potential clinical relevance and could help improve the knowledge of biomarkers in IPF patients. The PJ’s colonization role in IPF patients needs further research, and the complex interactions with the AKT/GSK-3β/snail pathway could explain some of our results. We showed that our analysis and findings are a valuable first approach to studying the host’s response to PJ, and it could open a new research line, which certainly deserves further exploration. An in-depth analysis of the underlined DEPs should be performed in future works to understand the underlying molecular networks, their complex mechanisms, and their emergent properties.

## Figures and Tables

**Table 1:** Characteristics of the study population.

Sample	Group	Label	Sex	Age
1	Control with PJ	114	Female	48
2	Control without PJ	115	Female	61
3	IPF with PJ	116	Male	60
4	IPF with PJ	116	Male	64
5	IPF with PJ	116	Male	76
6	IPF with PJ	116	Male	66
7	IPF with PJ	116	Male	67
8	IPF without PJ	117	Female	69
9	IPF without PJ	117	Male	45

PJ, *Pneumocystis jirovecii*; IPF, idiopathic pulmonary fibrosis.

**Table 2:** Summary of DEPs.

		Number of Proteins ^a^	
Group	Downregulated (<0.8)	Upregulated (>1.2)	Total
Control with PJ	38	33	71
IPF with PJ	33	35	68
IPF without PJ	27	34	61
Global			92

^a^ Fold change is the iTRAQ quantitative result. Fold change of <0.8 represents downregulation and fold change of >1.2 represents upregulation; PJ, *Pneumocystis jirovecii*; IPF, idiopathic pulmonary fibrosis.

**Table 3:** DEPs with opposite changes in control patients with PJ vs. IPF (with/without PJ) groups with significant *p*-values (*p* ≤ 0.05) in the three groups.

		Fold Change ^a^		
Protein Name	Gene Symbol	Control with PJ	IPF with PJ	IPF Without PJ
Vimentin	VIM	0.563	3.283	2.912
Hemoglobin subunit alpha	HBA1	6.184	0.559	0.729
Hemoglobin subunit beta	HBB	4.857	0.557	0.494

^a^ Fold change is the iTRAQ quantitative result. Fold change of <0.8 represents downregulation and fold change of >1.2 represents upregulation; PJ, *Pneumocystis jirovecii*; IPF, idiopathic pulmonary fibrosis.

## Data Availability

The original data presented in the study are openly available at https://github.com/IonasPi/Proteomic_Effect-of-P.-jirovecii-in-IPF (accessed on 25 January 2025).

## Supplementary Materials

The following supporting information can be downloaded at: https://www.mdpi.com/article/10.3390/jof11020102/s1.
